# Activating Transcription Factor 4 Promotes Esophageal Squamous Cell Carcinoma Invasion and Metastasis in Mice and Is Associated with Poor Prognosis in Human Patients

**DOI:** 10.1371/journal.pone.0103882

**Published:** 2014-07-31

**Authors:** Hongwu Zhu, Xiong Chen, Bin Chen, Bei Chen, Weibing Song, Dayong Sun, Yagang Zhao

**Affiliations:** 1 Department of Gastroenterology, Guangzhou General Hospital of the Guangzhou Military Command of the People's Liberation Army (PLA), Guangzhou, China; 2 Department of Oncology, Fuzhou General Hospital of the Nanjing Military Command of the PLA, Fuzhou, China; 3 Department of Oncology, Guangzhou General Hospital of the Guangzhou Military Command of the People's Liberation Army (PLA), Guangzhou, China; 4 Department of Gerontology, Guangzhou General Hospital of the Guangzhou Military Command of the People's Liberation Army (PLA), Guangzhou, China; Cincinnati Children's Hospital Medical Center, United States of America

## Abstract

**Background:**

Activating transcription factor 4 (ATF4) is a stress response gene that is involved in homeostasis and cellular protection. However, its expression and function in esophageal squamous cell carcinoma (ESCC) remains unknown. In this study, we aimed to determine the clinicopathologic significance of ATF4 in ESCC and its potential role in ESCC invasion and metastasis.

**Methodology/Principal Findings:**

We demonstrated that ATF4 overexpression is correlated with multiple malignant characteristics and indicates poor prognosis in ESCC patients. ATF4 expression was an independent factor that affected the overall survival of patients with ESCC after surgical resection. ATF4 promoted cell invasion and metastasis by promoting matrix metalloproteinase (MMP)-2 and MMP-7 expression, while its silencing significantly attenuated these activities both *in vitro* and *in vivo*.

**Conclusions/Significance:**

We report that ATF4 is a potential biomarker for ESCC prognosis and that its dysregulation may play a key role in the regulation of invasion and metastasis in ESCC cells. The targeting of ATF4 may provide a new strategy for blocking ESCC metastasis.

## Introduction

Esophageal cancer is the sixth leading cause of cancer-related mortality with nearly 407,000 deaths occurring worldwide each year [1]. Esophageal squamous cell carcinoma (ESCC) and esophageal adenocarcinoma (EA) are the main types of esophageal carcinoma; however, in the highest risk areas, such as Iran and China, 90% of cases involve squamous cell carcinomas [2]. In recent decades, the global incidence of ESCC has remained largely unchanged, whereas there has been a rapid increase in the incidence of EA in the USA and western Europe [3]. Although resection alone can be considered to be a potentially curative treatment for some esophageal cancer patients, its prognosis remains very poor, and the 5-year survival rate worldwide is less than 40% [4]. Therefore, many patients succumb to this disease as a result of metastasis and recurrence after surgical resection.

Although the clinical features, such as nodule number and size, cell type, tumor differentiation level, mitotic count, amount of perineural and lymphatic/vascular invasion, and T stage are related to the likelihood of survival after surgical resection [5], [6], [7], [8], [9], these factors are insufficient for differentiating patients who are at high risk for metastasis from those that are at low risk. Therefore, it is crucial to identify tumor molecular markers that are predictive of survival and metastasis and can identify ESCC patients who may benefit from surgical resection. Recently, several genes that are associated with ESCC metastasis have been identified, including Gadd45G [10], glioma-associated oncogene homolog 1 [11], lysine-specific demethylase 1 [12], maspin [13], PLCE1 [14], and CACNA2D3 [15]. These studies have indicated that specific genes may contribute to the metastasis of ESCC. However, the complex mechanisms that are involved in this process are far from being understood.

In mammalian cells, the eukaryotic translation initiation factor 2 α subunit (eIF2α) is phosphorylated by different eIF2α kinases in response to a variety of stress signals, including anoxia/hypoxia, endoplasmic reticulum stress, amino acid deprivation, and oxidative stress [16]. This phosphorylation event leads to a rapid decrease in global protein biosynthesis that is concurrent with the induced translation of genes that function to alleviate cellular damage from stress, including ATF4 [16], [17]. To metastasize, ESCC cells must successfully complete a series of events, including the invasion of the tumor cells into the surrounding tissues, the entry of the tumor cells into systemic circulation (intravasation), their survival during circulation, the extravasation of the cells to distant organs, and finally, the formation of secondary tumors [18]. During these events, the ESCC cells must avoid stress-associated cell death and thus are prompted to metastasize. Recently, the expression of ATF4 was found to be elevated in hypoxia-induced circulating tumor cells but not in parental cells [19]. In addition, hypoxia was found to stimulate the migration of breast cancer cells via the PERK/ATF4/LAMP3-arm of the unfolded protein response [20], suggesting a role of ATF4 in cancer metastasis. However, its expression and function in ESCC remains unknown.

In the present study, we have determined that ATF4 expression is frequently up-regulated in ESCC tissues compared with adjacent non-cancerous epithelial samples. Using a tissue microarray, we found that ATF4 overexpression correlated with the TNM stage and lymph node metastasis. In addition, positive ATF4 expression indicated poorer prognoses than negative ATF4 expression in patients with ESCC. Furthermore, we showed that ATF4 promoted the migration and invasion of ESCC cells both *in vitro* and *in vivo*. MMP-2 and MMP-7 are both essential for ATF4-induced ESCC cell invasion. Our findings highlight the importance of ATF4 dysfunction in promoting tumor progression and metastasis and implicate it as a potential therapeutic target for ESCC.

## Results

### ATF4 expression correlates with advanced clinical stage, lymph node metastasis, and poor prognosis in ESCC patients

**Figure 1:**
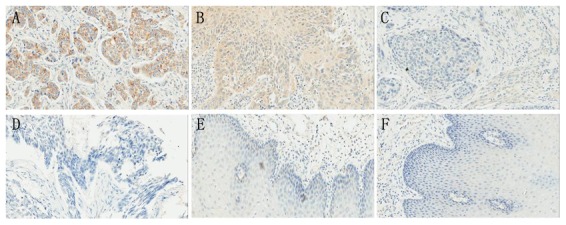
Representative IHC of ATF4 in matched ESCC and adjacent non-cancerous tissues. ESCC tissues with strong (A), moderate (B), weak (C), and negative (D) ATF4 staining. Adjacent non-cancerous tissues with weak (E) and negative (F) ATF4 staining. Magnification, 200×.

To determine whether ATF4 can be used as a predictive factor of the clinical outcomes of ESCC patients, immunohistochemistry was performed using 168 paraffin-embedded primary tumor samples and paired adjacent non-cancerous samples. Positive immunoreactivity for ATF4 was observed primarily in the cytoplasm of carcinoma cells and non-cancerous epithelial cells (Fig. 1). As summarized in Table 1, among all of the tumor samples that were analyzed, 30 (17.86%) demonstrated strong ATF4 staining (score of 9–12; Fig. 1A), 43 (25.60%) showed moderate staining (score of 5–8; Fig. 1B), 44 (26.19%) had weak staining (score of 2–4; Fig. 1C), and 51 (30.35%) exhibited negative staining (score of 0–1; Fig. 1D). In contrast, the majority of adjacent non-cancerous epithelial samples showed weak or negative staining for ATF4 (Fig. 1E and 1F).

**Table 1:** Differential ATF4 Expression in ESCC and Adjacent Non-Cancerous Tissues.

	Cases	ATF4 staining				*P* value
		−	+	++	+++	
ESCC tissues	168	51	44	43	30	<0.001
Adjacent non-cancerous tissues	168	114	41	9	4	

**Figure 2:**
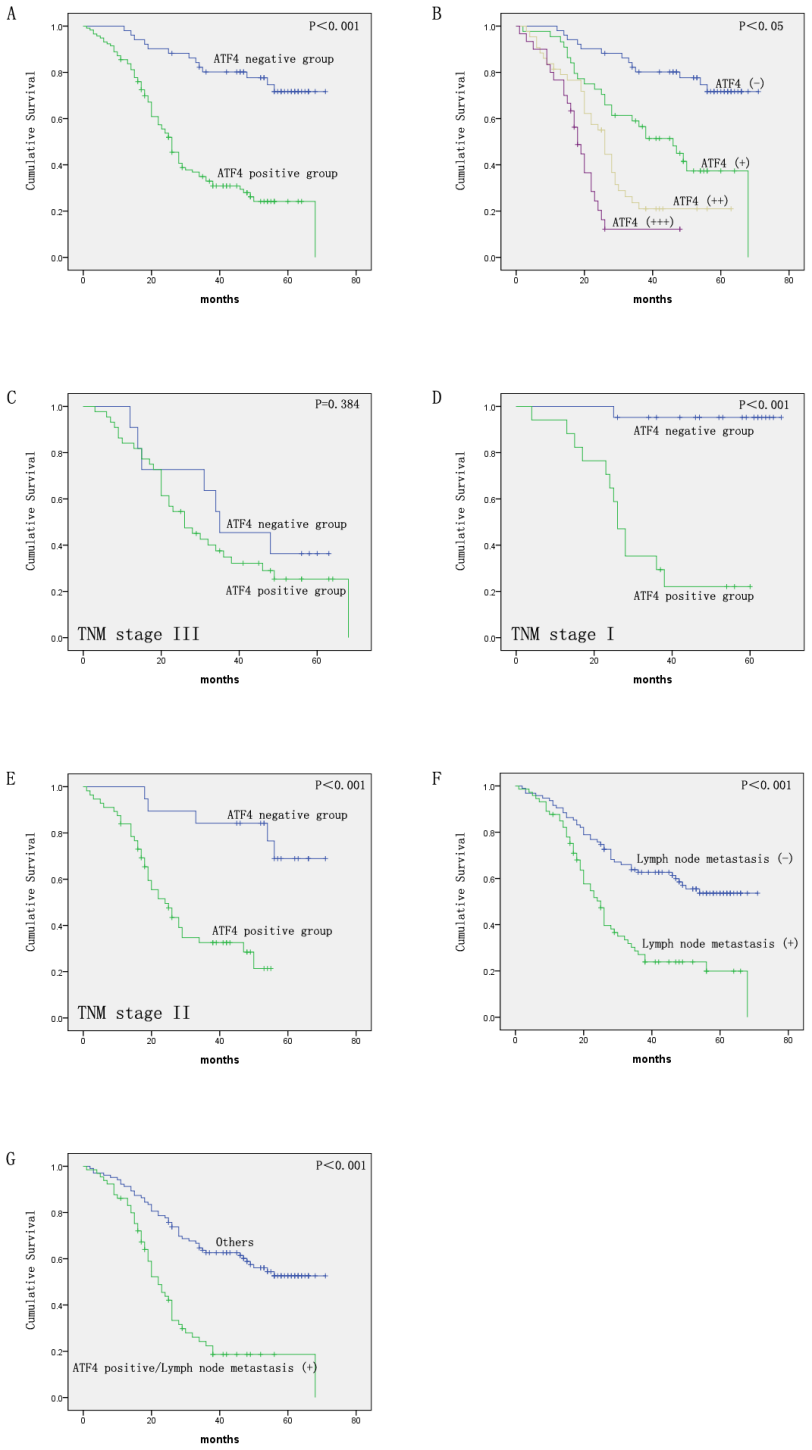
Kaplan-Meier survival curves of ESCC. Cases were stratified based on overall ATF4 expression levels (A) and ATF4 staining intensities (B). In the patient subgroups, cases were stratified based on overall ATF4 expression levels for the stage III (C), the stage I (D), and stage II (E) groups. (F) Cases were stratified based on lymph node metastasis status. (G) Cases stratified based on ATF4 expression (+) and lymph node metastasis (+).

Next, we explored the association between ATF4 protein expression and the clinicopathological characteristics of ESCC. The segregation of the patients into ATF4-positive and -negative groups did not reveal significant correlations with the clinicopathological parameters of age, sex, drinking habit, tumor site, or tumor differentiation. However, these groups did show significant correlations with TNM stage and lymph node metastasis (Table 2). Furthermore, we investigated the correlation of ATF4 expression with prognostic data. As a result, we found that the patients with ATF4-positive ESCC had significantly worse prognoses than those that were ATF4-negative. Additionally, the overall survival rates of ATF4-positive patients were significantly lower than those that were ATF4-negative (Fig. 2A; P<0.001). The staining intensity of ATF4 significantly correlated with shorter overall survival times (Fig. 2B). As shown in Table 3, univariate analyses showed that overall survival correlated with TNM stage, lymph node metastasis, and ATF4 expression. Furthermore, a multivariate Cox regression analysis indicated that ATF4 expression and lymph node metastasis were independent prognostic factors for overall survival (Table 3).

**Table 2:** Profiles of Patients with ATF4-Positive or ATF4-Negative ESCCs.

Clinicopathological Features	Tumor ATF4 Expression		*P* Value
	Negative(n = 51)	Positive(n = 117)	
Age	64.3(9.8)	63.9(9.7)	0.828
Sex			
Male	35	82	0.850
Female	16	35	
Drinking			
Yes	26	63	0.732
No	25	54	
Tumor site			
Upper	11	29	0.707
Middle	24	47	
Lower	16	41	
Tumor differentiation			
Well	26	53	0.549
Moderate	16	47	
Poor	9	17	
TNM stage			
I	21	17	0.001
II	19	56	
III	11	44	
Lymph node metastasis			
Absent	43	52	0.000
Present	8	65	

**Table 3:** Univariate and Multivariate Analysis of Factors Associated With Overall Survival of 168 ESCCs.

	Survival	
	Relative Risk (95% Confidence Interval)	*P* Value
Univariate analysis		
Age	1.004(0.982–1.026)	0.717
Sex(male/female)	0.749(0.474–1.183)	0.215
Drinking(yes/no)	0.856(0.570–1.285)	0.454
Tumor site(upper/middle/lower)	0.820(0.622–1.082)	0.161
Tumor differentiation(well/moderate/poor)	0.944(0.720–1.238)	0.679
TNM stage(I/II/III)	1.517(1.153–1.994)	0.003
Lymph node metastasis(absent/present)	2.604(1.724–3.934)	0.000
ATF4 expression(negative/positive)	4.935(2.722–8.946)	0.000
Multivariate analysis		
TNM stage(I/II/III)	1.118(0.834–1.500)	0.456
Lymph node metastasis(absent/present)	1.806(1.174–2.778)	0.007
ATF4 expression(negative/positive)	3.852(2.052–7.230)	0.000

The prognostic value of ATF4 protein expression in patient subgroups that were stratified according to tumor clinical stage was also analyzed. In the late stage group (III), no statistically significant differences were found in overall survival (P = 0.384; Fig. 2C), while in the stage I and II groups, the patients with positive ATF4 protein expression had significantly shorter overall survival rates compared with those with negative expression (P<0.001 for both; Fig. 2D and 2E). Thus, the level of ATF4 protein expression may be a valuable prognostic marker for patients with early- and mid-stage ESCC.

In previous studies, lymph node metastasis has been reported to be an important prognostic factor in patients with ESCC [21], [22]. Therefore, the correlation between lymph node metastasis and ESCC patient prognosis was also investigated in this study. The results showed that lymph node metastasis was significantly associated with a shorter overall survival time (P<0.001; Fig. 2F) and was identified to be an independent prognostic factor for overall survival by a multivariate analysis (P<0.001; Table 3). Consequently, a subset analysis was carried out by combining ATF4 expression with lymph node metastasis status. Our results demonstrated that patients with positive ATF4 expression/lymph node metastasis (+) had poorer overall survival rates compared with those of the other patients (P<0.001; Fig. 2G).

### Silencing of ATF4 inhibits migration, invasion, and metastasis of ESCC cells

**Figure 3:**
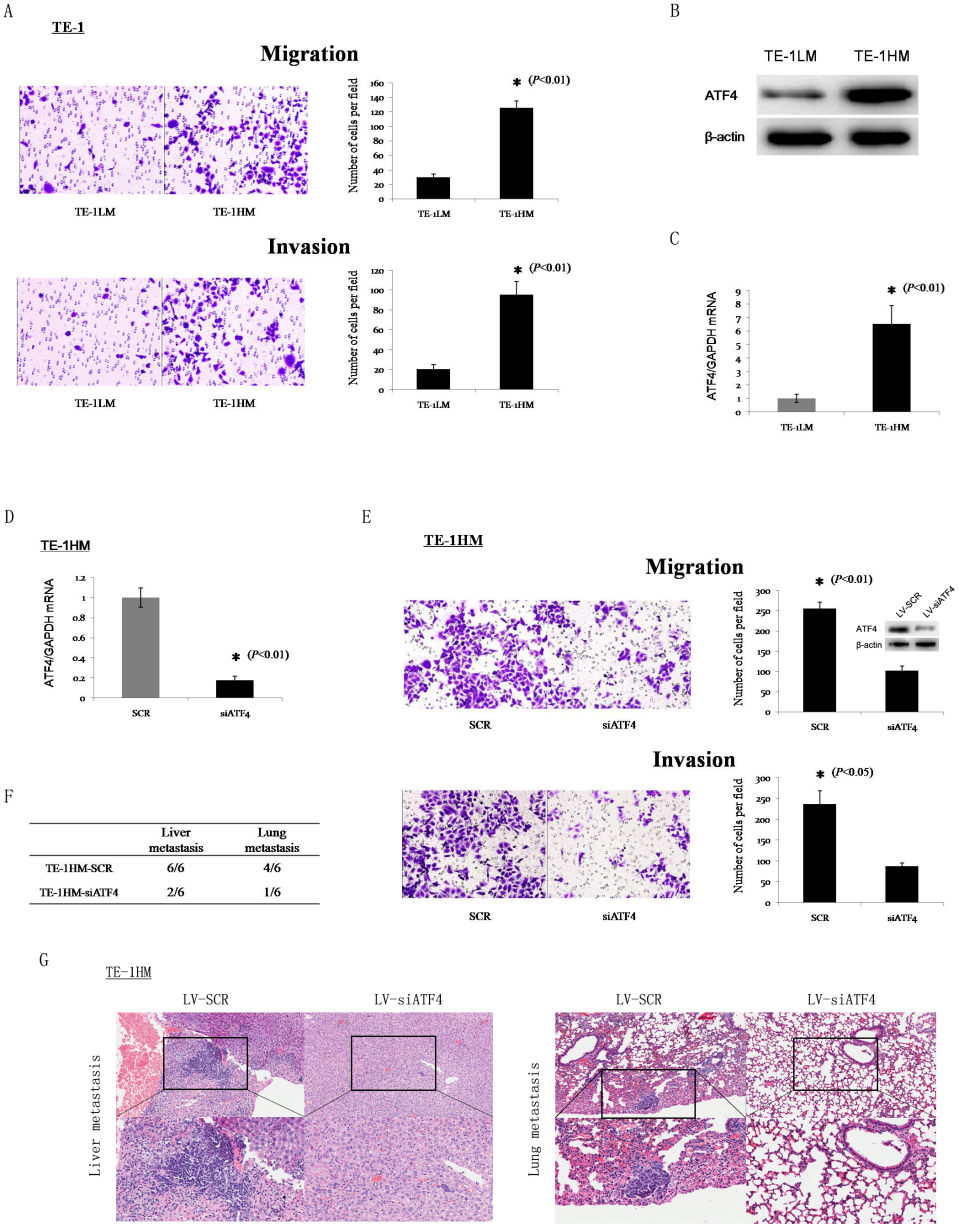
ATF4 silencing attenuates migration, invasion, and metastasis of ESCC cells. (A) The *in vitro* migration and invasion of the TE-1LM and TE-1HM cell sublines were examined by *in vitro* migration and invasion assays. Representative microscopic images of the underside of the transwell membrane are shown. Magnification, 200×. The bar graphs represent the average number of cells on the underside of the membrane ± S.D. The protein (B) and mRNA (C) levels of ATF4 in the TE-1LM and TE-1HM sublines were examined by Western blotting and qPCR. β-actin and GAPDH were used as internal controls, respectively. The data represent the means ± S.D. of three independent experiments. (D) ATF4 mRNA levels in the TE-1HM cells that were stably transfected with LV-SCR and LV-siATF4 were detected by qPCR. (E) *In vitro* migration and invasion assays of the TE-1HM cells that were stably transfected with LV-SCR and LV-siATF4. Inset, relative ATF4 protein expression levels as determined by Western blot. (F) Incidence of metastasis in mice implanted with TE-1HM-SCR or TE-1HM-siATF4 cells. (G) Representative hematoxylin and eosin staining of the lungs and livers of mice.

To establish models for the analysis of ESCC metastasis, we created invasive and non-invasive cell sublines from the human ESCC cell line TE-1 using the repeated transwell approach as previously described [23]. The transwell assay showed that the migration and invasion capacities of the TE-1HM cells (high metastatic potential) were significantly greater than those of the TE-1LM cells (low metastatic potential) (Fig. 3A). Next, the ATF4 levels in these two sublines were detected by Western blot and qPCR. Both the protein and mRNA levels of ATF4 were much higher in the TE-1HM cells than in the TE-1LM cells (Fig. 3B and 3C).

To determine the role of ATF4 in tumor metastasis, we knocked down ATF4 expression with the indicated lentiviral shRNA. Real-time PCR showed that the inhibitory efficiency of shRNA reached levels of 80% (Fig. 3D). In addition, ATF4 levels following the knockdown of specific shRNAs in the TE-1HM cells were slightly higher compared with the parental TE-1LM cells (Fig. S1). Subsequently, a transwell assay showed that the silencing of endogenous ATF4 expression in the TE-1HM cells significantly reduced cell migration (2.52-fold) and invasion (2.72-fold) (Fig. 3E). To further investigate the influence of ATF4 silencing on *in vivo* tumor metastasis, we injected TE-1HM-siATF4 or TE-1HM-SCR cells into nude mice through their tail veins. Most of the mice that were implanted with the TE-1HM-SCR cells showed liver and lung metastases, whereas less metastases were detected in the mice that had been implanted with the TE-1HM-siATF4 cells (Fig. 3F and 3G). Although the number of mice that were injected with the TE-1HM-siATF4 cells did not significantly differ from that of the mice that were injected with TE-1HM-SCR, when the presence of tumor nodules was macroscopically examined, the mice that had been implanted with the TE-1HM-SCR cells showed more liver and lung metastases compared with those that had been implanted with the TE-1HM-siATF4 cells (Fig. S2). This may be due to an insufficient number of mice in each group. Subsequently, the mice that were implanted with the TE-1HM-SCR cells did show statistically significant increases in liver and lung metastases compared with those that had been implanted with the TE-1HM-siATF4 cells following a repetition of the in vivo experiment using 10 mice per group (Table S1).

### Ectopic expression of ATF4 promotes ESCC cell migration, invasion, and metastasis *in vitro* and *in vivo*

**Figure 4:**
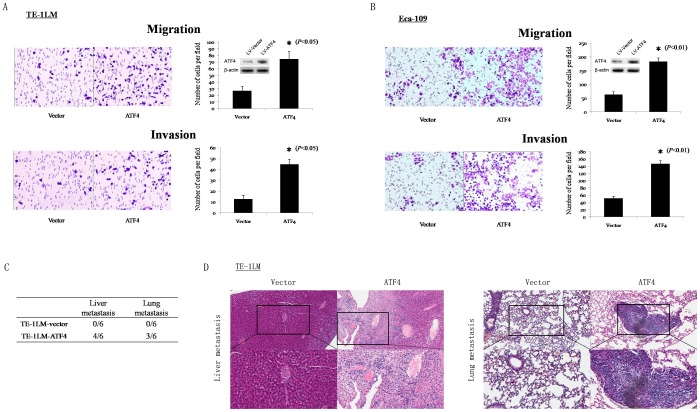
Overexpression of ATF4 promotes tumor cell invasion and metastasis. *In vitro* migration and invasion assays in TE-1LM (A) and Eca-109 (B) cells that were stably transfected with LV-vector or LV-ATF4. Inset, relative ATF4 protein expression levels as determined by Western blot. (C) Incidence of metastasis in mice implanted with TE-1LM-vector or TE-1LM-ATF4 cells. (D) Representative hematoxylin and eosin staining of the lungs and livers of mice.

The qPCR and Western blot analyses that were performed on the Eca-109, TE-1, TE-1LM, and TE-1HM cells showed that the ATF4 mRNA and protein levels increased progressively from the ESCC cells with low metastatic potential to those with high metastatic potential (Fig. S3). To further investigate the role of ATF4 in ESCC metastasis, ATF4-expression or control vectors were stably transfected into TE-1LM and Eca-109 cells. The *in vitro* assays revealed that the ectopic expression of ATF4 led to 2.80- and 3.53-fold increases in the migration and invasion of the TE-1LM cells, respectively (Fig. 4A). Similar results were observed in the Eca-109 cells in the *in vitro* migration (2.88-fold) and invasion (2.92-fold) assays (Fig. 4B). A tail vein assay of cancer metastasis was performed in nude mice to examine the *in vivo* metastatic abilities of the TE-1LM-ATF4 and TE-1LM-vector cells. ATF4 transfection led to significantly more liver and lung metastases compared with the empty vector-transfected control cells (Fig. 4C and 4D). Consistent with the afore mentioned results, ATF4 transfection led to similar findings compared with the empty vector-transfected Eca-109 cells (Table S2).

### ATF4 does not modulate proliferation or colony formation of ESCC cells

**Figure 5:**
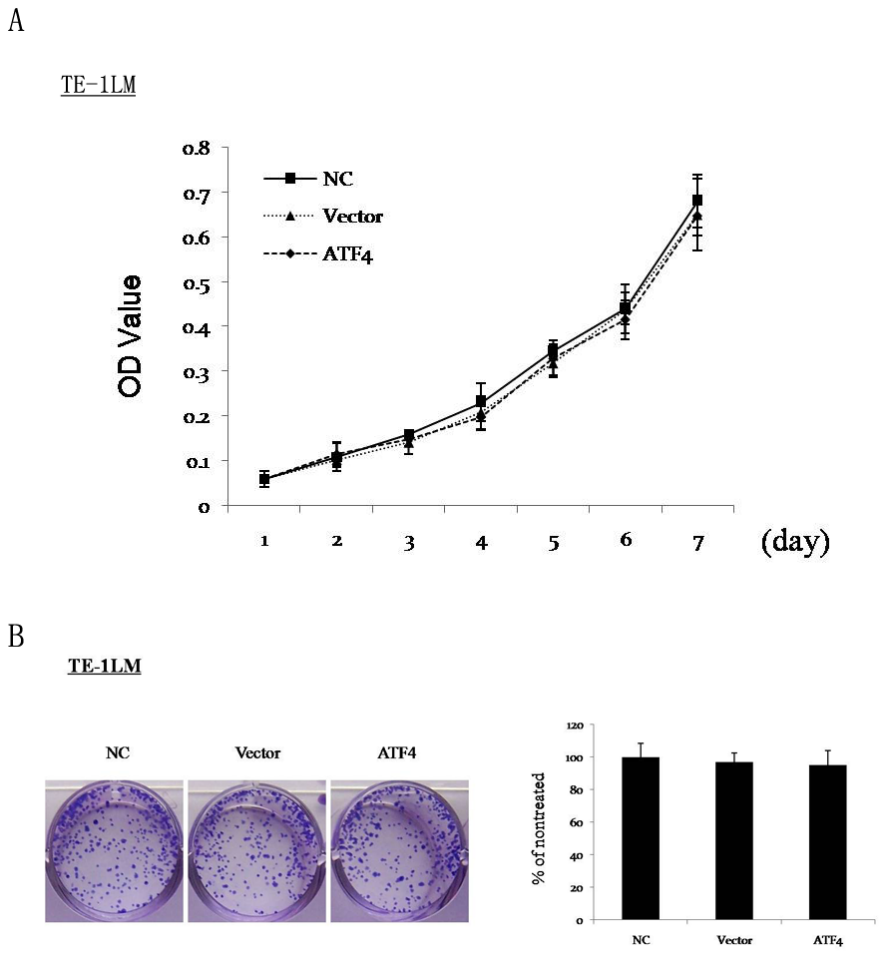
ATF4 does not affect the proliferation or colony formation of ESCC cells. (A) MTT assay of the proliferation of TE-1LM non-treated control (NC), TE-1LM-vector (Vector), and TE-1LM-ATF4 (ATF4) cells. The data represent the means ± S.D. of three independent experiments. (B) The effects of ATF4 on the proliferation of the cells as described above were also examined using a colony formation assay. The media was changed every 3 d. Cells were plated in triplicate, and the experiment was repeated three times. Representative wells are shown.

To investigate whether ATF4 modulates metastasis by affecting ESCC cell proliferation, we examined the proliferation of ESCC cells with or without ATF4 transfection. In fact, no statistically significant differences were found between the TE-1LM-vector cells and TE-1LM-ATF4 cells (Fig. 5A). Similarly, ATF4 did not modulate the proliferation of Eca-109 cells (data not shown). Furthermore, a colony formation assay indicated that there were no statistically significant differences in colony numbers between the ATF4- and control vector-transfected TE-1LM cells (Fig. 5B).

These observations indicate that ATF4 confers a metastatic phenotype to ESCC cells without affecting their proliferation or colony formation abilities.

### MMP-2 and MMP-7 are involved in ATF4-mediated tumor invasion and metastasis

**Figure 6:**
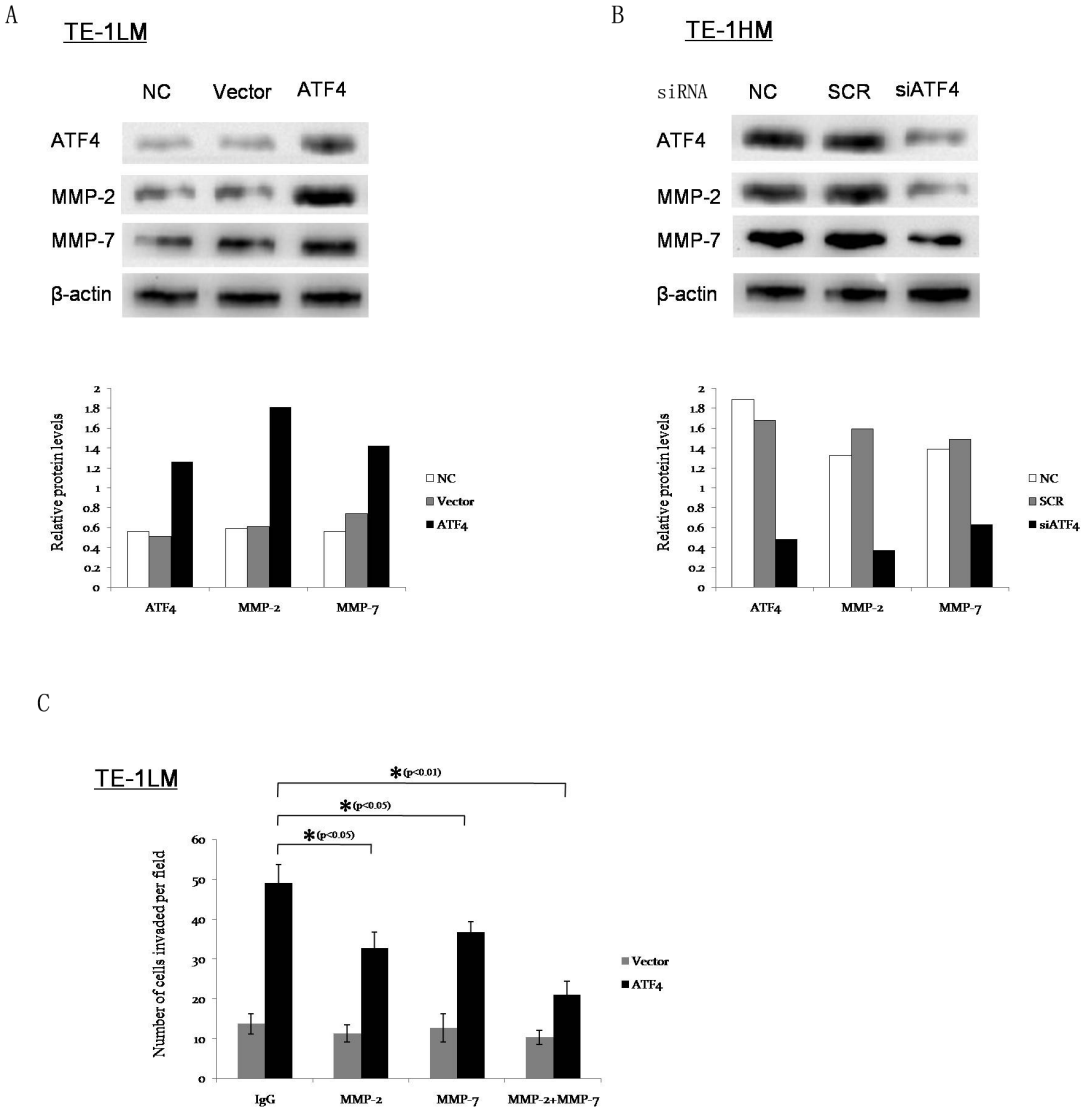
MMP-2 and MMP-7 are associated with and essential for ATF4-mediated tumor invasion and metastasis. (A) The protein levels of MMP-2 and MMP-7 in the TE-1LM-vector and TE-1LM-ATF4 cells were examined by Western blotting. β-actin was used as an internal control. The relative protein/β-actin ratio is shown (bottom). (B) The protein levels of MMP-2 and MMP-7 in the TE-1HM-SCR and TE-1HM-siATF4 cells were examined by Western blotting. The relative protein/β-actin ratio is shown (bottom). (C) The inhibition of MMP-2 and MMP-7 using neutralizing antibodies decreased ATF4-enhanced cell invasion. The TE-1LM-vector and TE-1LM-ATF4 cells were preincubated with the indicated antibody (2 µg/ml) for 24 h. Then, the invasive ability of the cells was examined using an *in vitro* invasion assay. During the assay, the cells were treated continuously with the indicated antibody. The data represent the means ± S.D. of three independent experiments.

Extracellular matrix (ECM) degradation is an essential step in tumor invasion and metastasis, which is mainly mediated by MMPs [24]. To determine whether ATF4 facilitates ESCC cell invasion by regulating MMP expression, we examined its effect on the expression of several MMPs in TE-1LM cells after transfection. Western blot analyses showed that MMP-2 and MMP-7 were significantly up-regulated by ATF4 (Fig. 6A), whereas it did not significantly affect the expression of the other studied MMPs (data not shown). In contrast, the siRNA knockdown of ATF4 in TE-1HM cells resulted in the significantly reduced endogenous expression of MMP-2 and MMP-7 (Fig. 6B).

To study the possible roles of MMP-2 and MMP-7 in ATF4-enhanced cell invasion, TE-1LM-ATF4 cells were treated with MMP-2- or MMP-7-neutralizing antibodies. As shown in Fig. 6C, both of the antibodies significantly reduced ATF4-enhanced cell invasion, and in combination, these antibodies worked synergistically to maximally reverse the invasion phenotypes of the TE-1LM-ATF4 cells.

To investigate whether MMP-2 and MMP-7 were up-regulated by ATF4 *in vivo*, we performed immunohistochemistry in the metastatic tumors that had been injected with the TE-1LM-ATF4, TE-1HM-SCR, and TE-1HM-siATF4 cells. Among all of the tumor samples that were analyzed, tumors from the TE-1LM-ATF4 and TE-1HM-SCR cell groups showed strong and moderate MMP-2 and MMP-7 staining (Fig. S4). In contrast, those from the TE-1HM-siATF4 group showed weak or negative staining (Fig. S4). These results indicate that ATF4 promotes ESCC cell invasion and metastasis by promoting MMP-2 and MMP-7 expression *in vivo*. Furthermore, immunohistochemistry also showed that ATF4 expression positively correlated with MMP-2 and MMP-7 expression in the ESCC tissues (Fig. S5).

Taken together, these results suggest that MMP-2 and MMP-7 are involved in and essential for ATF4-mediated tumor invasion and metastasis.

## Discussion

In the present study, we showed that ATF4 is frequently up-regulated in ESCC tissues compared with non-cancerous epithelial samples. In addition, clinical evidence indicated that ATF4 overexpression correlates with TNM stage and lymph node metastasis. Furthermore, the Kaplan-Meier analysis showed that the patients with ESCC and positive ATF4 expression had significantly worse prognoses than those with negative ATF4 expression. A multivariate analysis revealed that ATF4 expression was an independent prognostic factor for survival after surgical resection. These clinical data strongly suggest that ATF4 contributes to the progression and metastasis of ESCC.

Emerging but limited evidence suggests that ATF4 regulates the metastasis of tumor cells. It has been reported that the induction of the metastasis-associated gene LAMP3 occurs as a consequence of the activation of the PERK/eIF2α/ATF4-arm of the unfolded protein response (UPR) and is independent of HIF-1α [25]. Additionally, hypoxia-induced ATF4 has been found in circulating tumor cells (high metastatic potential) but not in their parental cells [19]. In breast cancer, the PERK/ATF4/LAMP3-arm of the UPR is an additional pathway mediating hypoxia-induced tumor cell migration [20]. In this study, our *in vitro* and *in vivo* data demonstrate that the overexpression of ATF4 promotes the migration and invasion of ESCC cells with low metastatic potential, while the silencing of ATF4 suppresses the migration and invasion of ESCC cells with high metastatic potential. Thus, these data suggest that ATF4 is an important downstream mediator of metastasis that functions via multiple mechanisms and is, therefore, a valuable therapeutic target.

Tumor cells must successful undergo a series of sequential and selective events to metastasize, including detachment, migration, local invasion, intravasation, survival in the circulatory system, extravasation, and regrowth in distant metastatic organs [26]. In fact, degradation of the ECM is an essential step in tumor invasion and metastasis, which is mainly regulated by MMPs [24]. In this study, we revealed that ATF4 promotes ESCC cell migration and invasion *in vitro* and liver and lung metastases *in vivo*. The inhibition of endogenous ATF4 expression significantly reduced the migratory and invasive abilities of the ESCC cells *in vitro* in addition to the incidence of liver and lung metastases *in vivo*. In addition, we found that the expressions of MMP-2 and MMP-7 were up-regulated in the ATF4-overexpressing cells. The treatment of ESCC cells with MMP-2- or MMP-7-neutralizing antibodies significantly reduced ATF4-enhanced cell invasion. Furthermore, immunohistochemistry showed that ATF4 expression was positively correlated with MMP-2 and MMP-7 expression in the ESCC tissues. These results indicate that MMP-2 and MMP-7 are both essential for ATF4-induced cell invasion, although whether they are direct or indirect transcriptional targets of ATF4 remains to be elucidated.

In conclusion, we have demonstrated that ATF4 overexpression is associated with metastatic characteristics and poor prognosis in ESCC patients. It promotes ESCC cell invasion and metastasis by up-regulating the expression of MMP-2 and MMP-7. Moreover, ATF4 is a valid target in metastatic ESCC cells, and the development of effective ATF4 inhibitors should be taken into consideration in the future. These findings provide novel insight into the role of ATF4 in tumorigenesis and metastasis. This is especially important for clinical consideration because ATF4 can be up-regulated by oxygen deprivation, oxidative stress, nutritional deprivation, and almost all of the adverse stressors in the tumor microenvironment, which could be hijacked by cancer cells to promote invasion and metastasis. Therefore, interventions that are based on disrupting stress-induced ATF4 expression in cancer cells may be helpful in developing effective treatments of esophageal squamous cell carcinoma.

## Materials and Methods

### Ethics statement

All of the experimental procedures were approved by the Institutional Review Board of the Guangzhou General Hospital of the Guangzhou Military Command of the PLA. Written informed consent was obtained for all of the patient samples. All of the procedures involving animal experimentation were performed according to the guidelines of the National Institutes of Health Guide for the Care and Use of Laboratory Animals and were approved by the Animal Care and Use Committee of the Guangzhou General Hospital of the Guangzhou Military Command of the PLA. All efforts were made to minimize the number of animals that were used and their suffering.

### Patients and clinical specimens

The tissue samples that were used to create the tissue arrays were obtained from 168 patients with esophageal cancer who had undergone surgery at the Guangzhou General Hospital of the Guangzhou Military Command of the PLA between 2006 and 2012. None of the patients received preoperative chemotherapy or radiotherapy. All of the cases of esophageal cancer and adjacent non-cancerous tissues were validated clinically and pathologically. The clinicopathological characteristics are shown in Table 2. All of the patients were followed until the end of 2012.

### Immunohistochemistry

Immunohistochemistry was performed as described elsewhere [27]. Dewaxed and rehydrated slides were washed in fresh water for 10 min and then incubated in 10% normal bovine serum for 1 h. Next, the slides were incubated with a polyclonal anti-ATF4 antibody (ab31390, 1∶100 dilution; Abcam, Cambridge, UK), anti-MMP-2 antibody (bs-0412R, 1∶200 dilution; Biosynthesis Biotechnology Co., Beijing, China), and anti-MMP-7 antibody (bs-0423R, 1∶200 dilution; Biosynthesis Biotechnology Co., Beijing, China) at 4°C overnight in a moist chamber. The slides were sequentially incubated with biotinylated goat anti-rabbit IgG (Zhongshan Goldenbridge Biotechnology, Wuhan, China) and then peroxidase-conjugated streptavidin, each for 30 min at room temperature. Isotope-matched human IgG was used in each case as a negative control. Finally, the 3, 5-diaminobenzidine (DAB) Substrate Kit (Zhongshan Goldenbridge Biotechnology, Wuhan, China) was used for color development, followed by counterstaining with Mayer's hematoxylin. All of the slides were examined independently by two experienced pathologists who were blinded to the clinicopathological information. The average value of the two independent scores is shown. The expression of ATF4 was evaluated by examining the proportion of positive cells per specimen and the staining intensity. The ratio of positive cells was calculated by comparing the number of stained tumor cells to the total number of tumor cells as follows: 0, staining of ≤1%; 1, staining of 2–25%; 2, staining of 26–50%; 3, staining of 51–75%; and 4, staining of >75%. The staining intensity was graded as follows: 0, achromatic; 1, amber; 2, yellow; and 3, brown. A total score of 0–12 was finally calculated asthe ratio of positively stained cells (score)×intensity of immunoreactivity (score). The scores were graded as negative (−; score 0–1), weak (+; score 2–4), moderate (++; score 5–8), and strong (+++; score 9–12).

### Cell culturing and isolation of sublines with different metastatic potentials

The human ESCC cell lines TE-1 and Eca-109 (obtained from the cell bank of the Chinese Academy of Sciences, Shanghai, China) were cultured in RPMI-1640 medium that was supplemented with 10% fetal bovine serum (Hyclone, Thermo Scientific, US) and penicillin/streptomycin at 37°C and 5% CO_2_ in a humidified incubator.

ESCC cell sublines with different metastatic potentials were isolated as described elsewhere [23]. We created two ESCC cell sublines with either low or high metastatic potential from the human ESCC cell line TE-1 using the repeated transwell approach. After ten rounds of selection, we obtained an ESCC cell line with low metastatic potential (designated as TE-1LM) and one with high metastatic potential (designated as TE-1HM).

### Plasmid construction and cell transfection

The 1056-bp human ATF4 coding sequence was synthesized and subcloned into the NheI and HindIII sites of the pcDNA3.1 vector. The pcDNA3.1-ATF4 plasmid was validated by sequencing. The lentiviral vectors were constructed as described previously [28]. The stable cell lines were generated by the transfection of cells with the indicated lentiviral constructs followed by their selection in puromycin or zeocin (Invitrogen), respectively.

### Immunoblotting

Cells were washed with cold phosphate-buffered saline (PBS) and lysed. The protein concentration of the cell lysate was determined using the Bradford reagent (Bio-Rad) with BSA as a standard. An equal amount of protein for each sample was loaded, separated by SDS-PAGE, and transferred onto a 0.22-µm nitrocellulose membrane (Millipore). The blots were probed with anti-ATF4 (sc-200, Santa Cruz), anti-MMP-1 (sc-21731, Santa Cruz), anti-MMP-2 (sc-13594, Santa Cruz), anti-MMP-7 (sc-8832, Santa Cruz), anti-MMP-9 (sc-21733, Santa Cruz), anti-MMP-13 (sc-30073, Santa Cruz), and anti-β-actin (sc-47778, Santa Cruz). The bound antibodies were visualized using horseradish peroxidase (HRP)-conjugated secondary antibodies (Santa Cruz) and an enhanced chemiluminescent reagent (Pierce). Quantification was performed using the Quantity One software (Bio-Rad).

### Quantitative real-time PCR (qPCR)

Total RNA was isolated with the Qiagen RNeasy Kit (Qiagen). The DNase I treatment was included before the final elution to eliminate genomic DNA contamination. The cDNA was reverse transcribed from 1.0 mg of total RNA with random 6-mers and an oligo dT Primer using the PrimeScript RT reagent Kit (TaKaRa) according to the manufacturer's instructions. Quantitative real-time PCR was performed on equal volumes of cDNA using a LightCycler 480 II System (Roche) and SYBR Green PCR Master Mix (TaKaRa). The following primers were used for the qPCR analysis: glyceraldehyde-3-phosphate dehydrogenase (GAPDH), 5′-CGG AGT CAA CGG ATT TGG TCG TAT-3′ and 5′-AGC CTT CTC CAT GGT GGT GAA GAC-3′; ATF4, 5′-TCA AAC CTC ATG GGT TCT CC-3′ and 5′-GTG TCA TCC AAC GTG GTC AG-3′.

### *In vitro* migration and invasion assay

The *in vitro* migration and invasion assay was performed as described elsewhere [23] with slight modifications. A 24-well transwell plate (Millicell PI8P01250, Millipore) was used to measure the migratory and invasive abilities of each cell line. For the migration assay, 2.5×10^4^ cells were plated in the top chamber of the transwell, which was lined with a non-coated membrane. For the invasion assay, the chamber inserts were coated with 200 mg/ml Matrigel (BD Biosciences, US) and dried overnight under sterile conditions, and then 5×10^4^ cells were plated in the top chamber. In both assays, the cells were suspended in medium without serum or growth factors, and medium that was supplemented with serum was used as a chemoattractant in the lower chamber. After incubation at 37°C for 24 h, the top chambers were wiped with cotton wool to remove any non-migratory or non-invasive cells. The invading cells on the underside of the membrane were fixed in 100% methanol for 10 min, air-dried, stained with 0.1% crystal violet, and counted under a microscope. The mean of triplicate assays for each experimental condition was calculated.

### *In vivo* metastasis assay

The *in vivo* metastasis assay was performed as described elsewhere [29] with slight modifications. BALB/C-nu/nu nude mice (5 weeks old) were used, and each experimental group consisted of 6 mice. Briefly, 1×10^6^ cells were injected intravenously through the tail vein of each mouse. All of the mice were euthanized at 6 weeks after the injection. The presence of tumor nodules was macroscopically determined. The tumor tissues were dissected from the liver and lung and examined histologically.

### Statistical analyses

For the comparisons of the data, the χ^2^ test, Fisher's exact test, one-way analysis of variance, and two-tailed Student's t-test were performed as appropriate. The overall survival probability was evaluated using the Kaplan-Meier method, and differences were assessed using the log-rank test. The Cox multivariate regression analysis was used to determine the independent prognostic factors. All of the statistical analyses were performed using the SPSS16.0 software (Chicago, IL). The significance level was set at 5%.

## Supporting Information

Figure S1 **ATF4 expression in TE-1-derived cell lines.** The protein (A) and mRNA (B) levels of ATF4 in the TE-1HM-SCR, TE-1HM-siATF4, and TE-1LM cells were examined by Western blotting and qPCR. β-actin and GAPDH were used as internal controls, respectively. The data represent the means ± S.D. of three independent experiments.
(TIF)

Figure S2 **Number of metastatic tumors macroscopically examined in mice implanted with TE-1HM-SCR and TE-1HM-siATF4 cells.** The *in vivo* experiment was performed as described in the **Materials and Methods** section with 6 mice per group. After all of the mice were euthanized, the presence of tumor nodules was macroscopically determined.
(TIF)

Figure S3 **ATF4 expression in ESCC cell lines with different metastatic potentials.** The protein (A) and mRNA (B) levels of ATF4 in the Eca-109, TE-1, TE-1LM, and TE-1HM cells were examined by Western blotting and qPCR. β-actin and GAPDH were used as internal controls, respectively. The data represent the means ± S.D. of three independent experiments.
(TIF)

Figure S4 **Representative IHC of ATF4, MMP-2, and MMP-7 in metastatic tumors in liver tissues of nude mice.** IHC for ATF4, MMP-2, and MMP-7 in the metastatic tumors, which were injected with TE-1LM-ATF4, TE-1HM-SCR, and TE-1HM-siATF4 cells. Representative images from each cohort are shown. Magnification, 200×.
(TIF)

Figure S5 **ATF4 expression was positively correlated with MMP-2/MMP-7 expression in ESCC tissues.** (A) IHC for ATF4, MMP-2, and MMP-7 in ESCC tissues. Representative images are shown. Magnification, 200×. (B) The correlation between the expression of ATF4 and MMP-2 or MMP-7.
(TIF)

Table S1 **Incidence of metastasis in mice implanted with TE-1HM-SCR and TE-1HM-siATF4 cells.**
(DOCX)

Table S2 **Incidence of metastasis in mice implanted with Eca-109-Vector and Eca-109-ATF4 cells.**
(DOCX)
