# Supplementary figures and images for: Activating Transcription Factor 4 Promotes Esophageal Squamous Cell Carcinoma Invasion and Metastasis in Mice and Is Associated with Poor Prognosis in Human Patients
Source: PLoS One. 2014 Jul 31;9(7):e103882. doi: 10.1371/journal.pone.0103882 (PMC4117569; doi:10.1371/journal.pone.0103882)

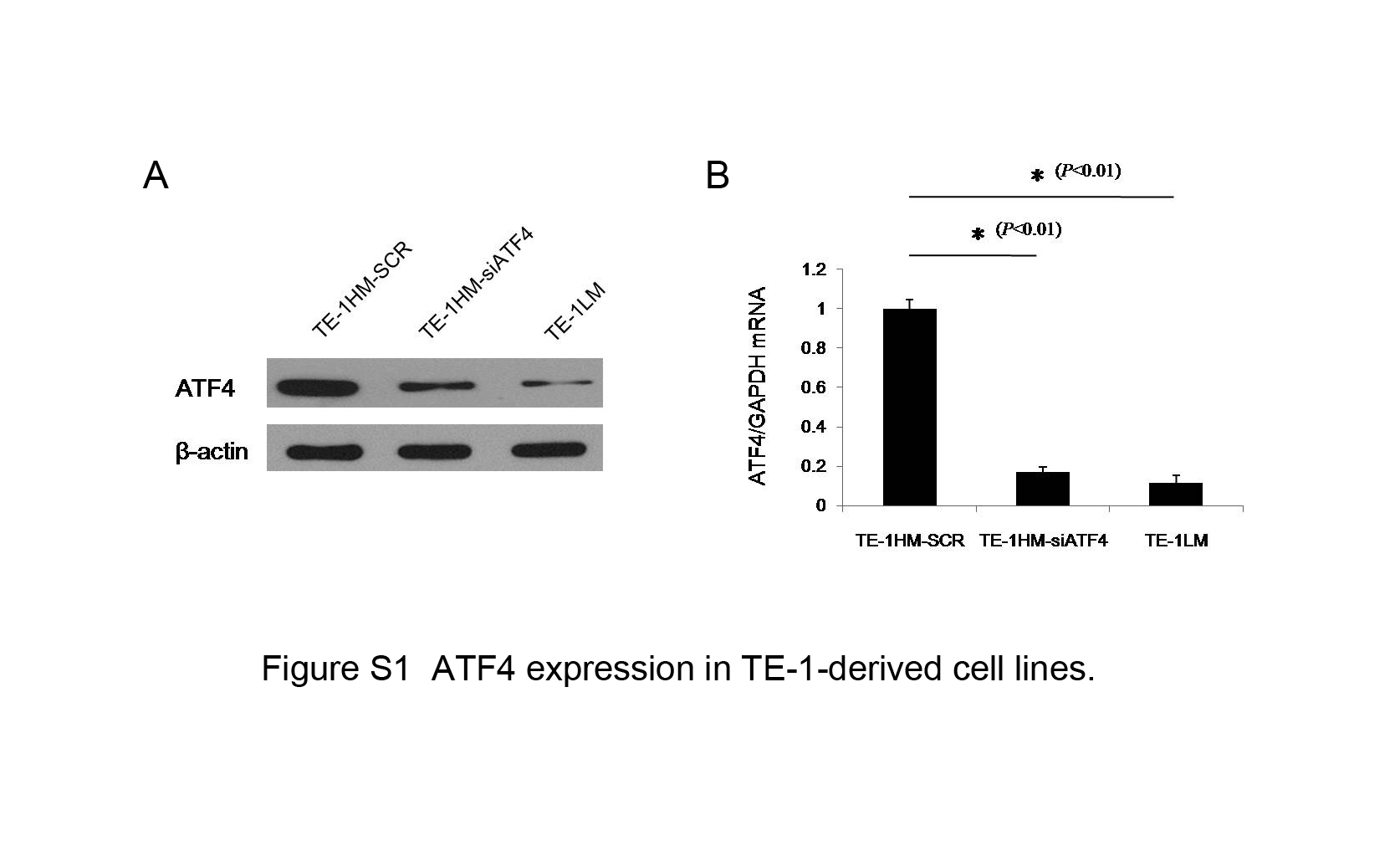

Supplement: Figure S1 — ATF4 expression in TE-1-derived cell lines. The protein (A) and mRNA (B) levels of ATF4 in the TE-1HM-SCR, TE-1HM-siATF4, and TE-1LM cells were examined by Western blotting and qPCR. β-actin and GAPDH were used as internal controls, respectively. The data represent the means ± S.D. of three independent experiments. (TIF) [file pone.0103882.s001.tif]

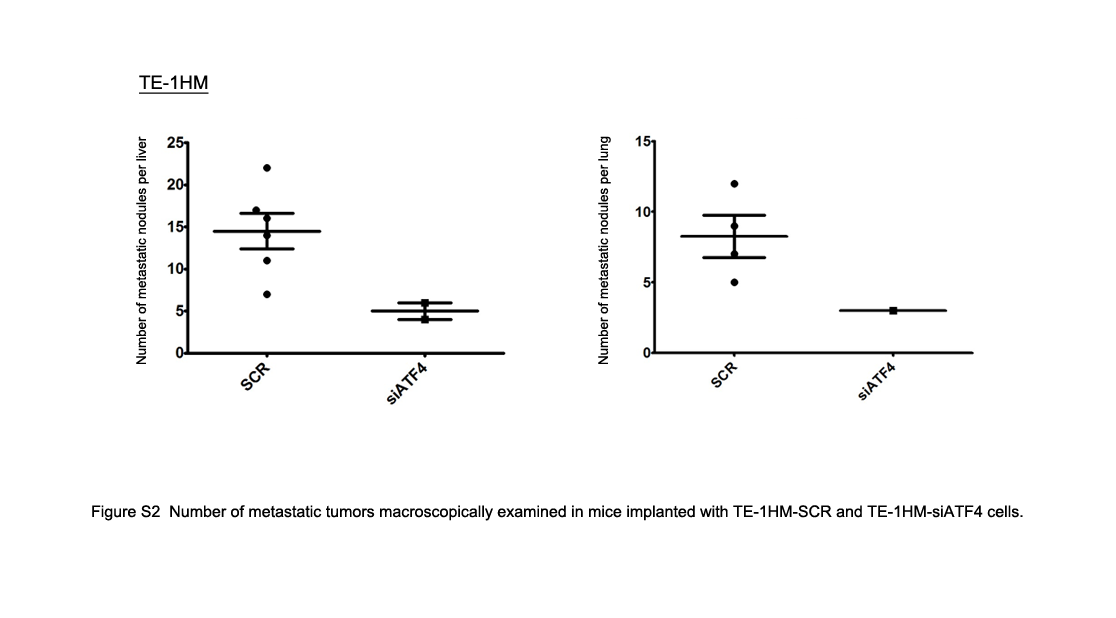

Supplement: Figure S2 — Number of metastatic tumors macroscopically examined in mice implanted with TE-1HM-SCR and TE-1HM-siATF4 cells. The in vivo experiment was performed as described in the Materials and Methods section with 6 mice per group. After all of the mice were euthanized, the presence of tumor nodules was macroscopically determined. (TIF) [file pone.0103882.s002.tif]

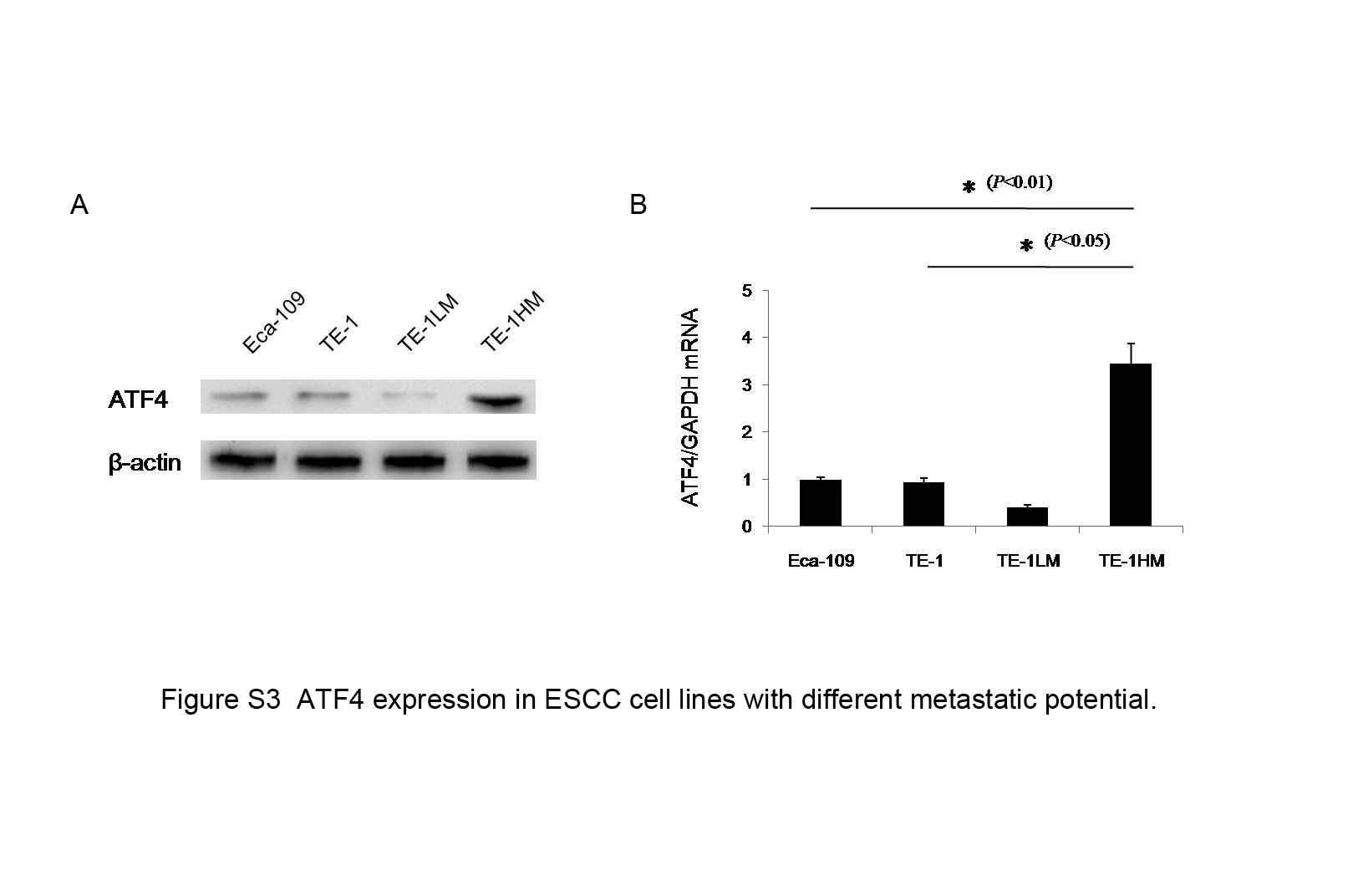

Supplement: Figure S3 — ATF4 expression in ESCC cell lines with different metastatic potentials. The protein (A) and mRNA (B) levels of ATF4 in the Eca-109, TE-1, TE-1LM, and TE-1HM cells were examined by Western blotting and qPCR. β-actin and GAPDH were used as internal controls, respectively. The data represent the means ± S.D. of three independent experiments. (TIF) [file pone.0103882.s003.tif]

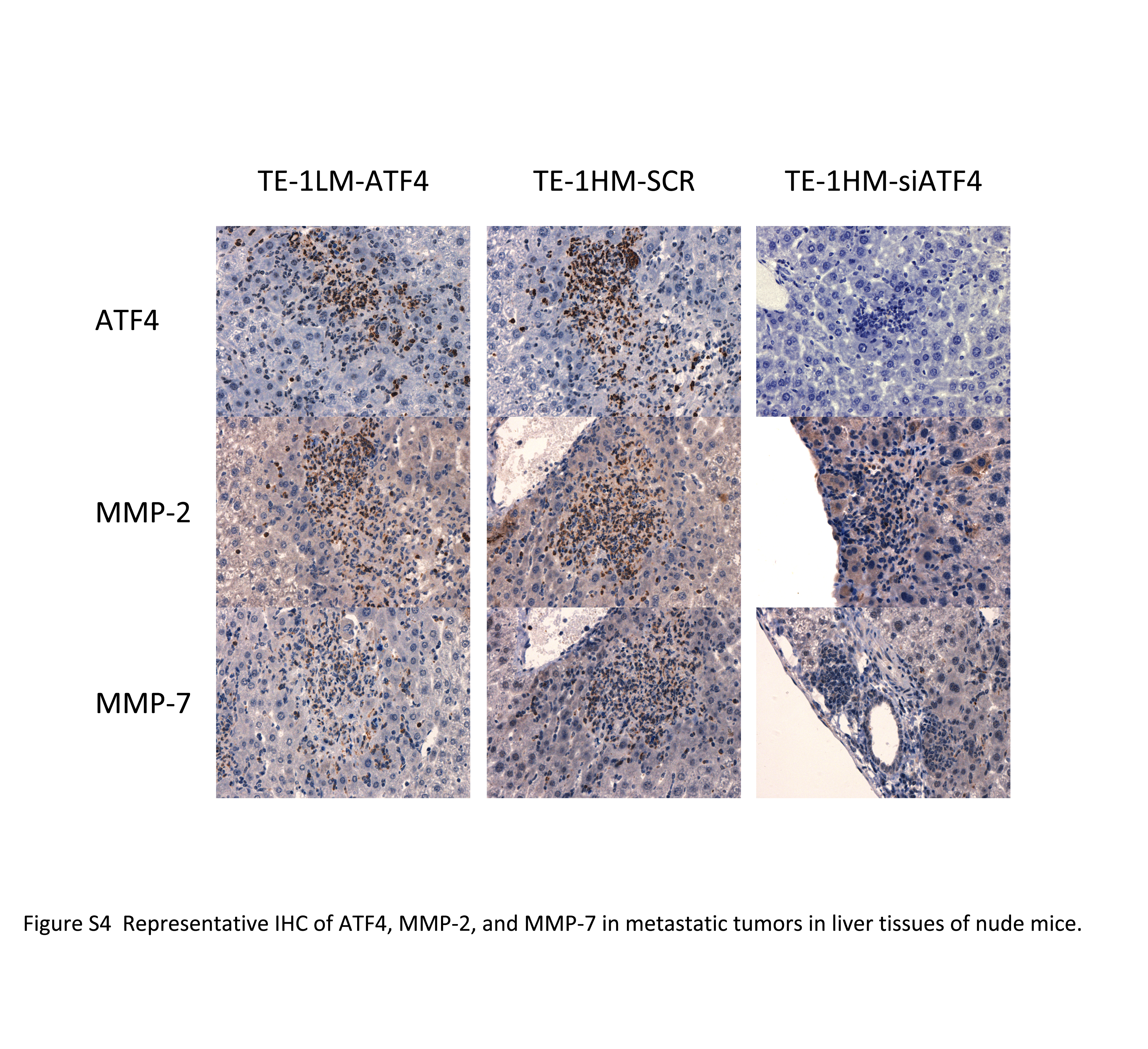

Supplement: Figure S4 — Representative IHC of ATF4, MMP-2, and MMP-7 in metastatic tumors in liver tissues of nude mice. IHC for ATF4, MMP-2, and MMP-7 in the metastatic tumors, which were injected with TE-1LM-ATF4, TE-1HM-SCR, and TE-1HM-siATF4 cells. Representative images from each cohort are shown. Magnification, 200×. (TIF) [file pone.0103882.s004.tif]

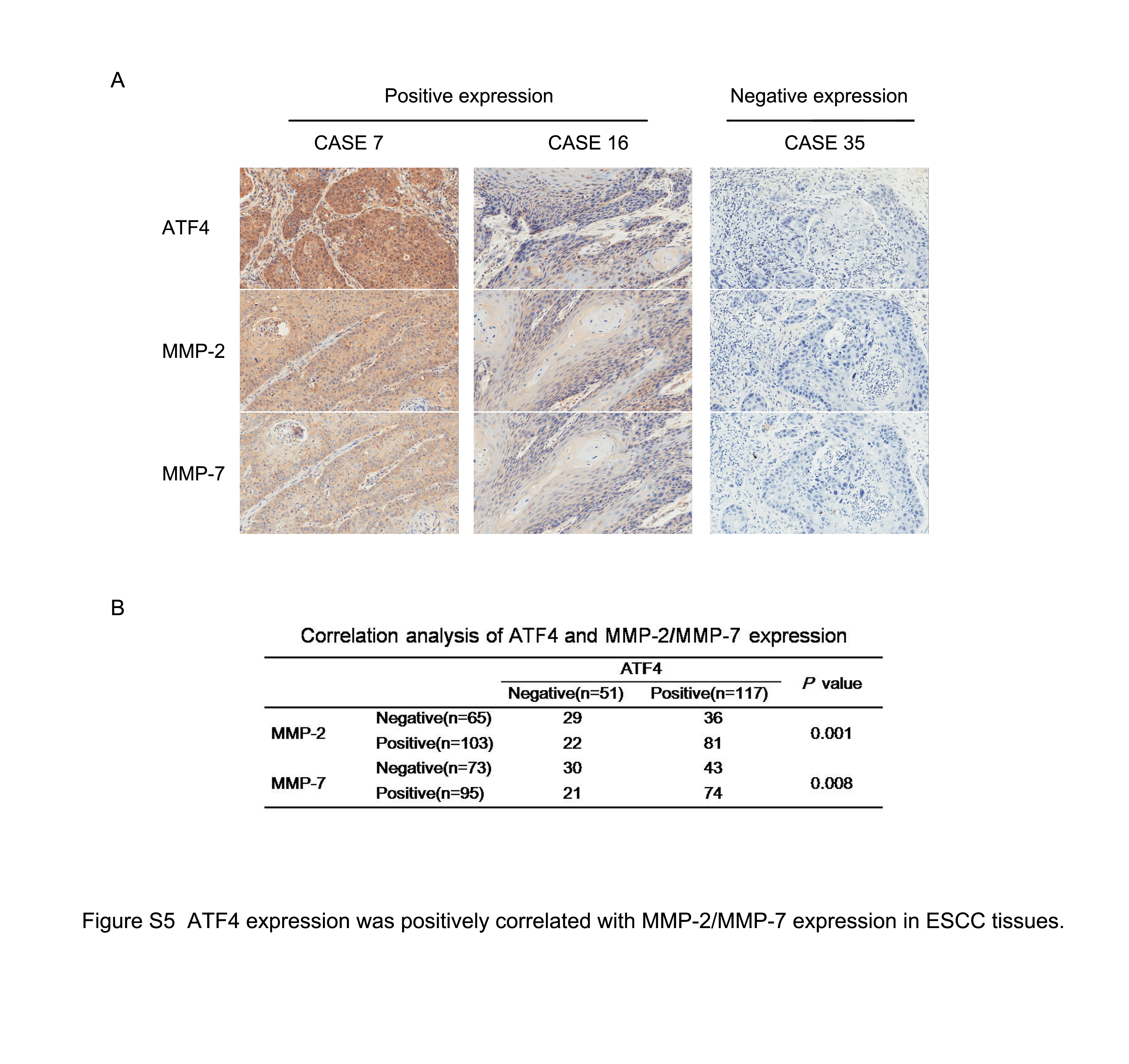

Supplement: Figure S5 — ATF4 expression was positively correlated with MMP-2/MMP-7 expression in ESCC tissues. (A) IHC for ATF4, MMP-2, and MMP-7 in ESCC tissues. Representative images are shown. Magnification, 200×. (B) The correlation between the expression of ATF4 and MMP-2 or MMP-7. (TIF) [file pone.0103882.s005.tif]
