# Supplementary material for: Activating Transcription Factor 4 Promotes Esophageal Squamous Cell Carcinoma Invasion and Metastasis in Mice and Is Associated with Poor Prognosis in Human Patients
Source: PLoS One. 2014 Jul 31;9(7):e103882. doi: 10.1371/journal.pone.0103882 (PMC4117569; doi:10.1371/journal.pone.0103882)
Supplement: Table S2 — Incidence of metastasis in mice implanted with Eca-109-Vector and Eca-109-ATF4 cells. (DOCX) [file pone.0103882.s007.docx]

Table S2. Incidence of metastasis in mice implanted with Eca-109-Vector and Eca-109-ATF4 cells

|  | Eca-109-Vector | Eca-109-ATF4 | *P* value |
| --- | --- | --- | --- |
| Liver metastasis | 3/10 | 8/10 | <0.05 |
| Lung metastasis | 2/10 | 7/10 | <0.05 |
